# Manipulation of pore structure during manufacture of agarose microspheres for bioseparation

**DOI:** 10.1002/elsc.202000023

**Published:** 2020-09-23

**Authors:** Lan Zhao, Yongdong Huang, Kai Zhu, Zhuang Miao, Jiazhang Zhao, Xiang Jing Che, Dongxia Hao, Rongyue Zhang, Guanghui Ma

**Affiliations:** ^1^ State Key Laboratory of Biochemical Engineering Institute of Process Engineering Chinese Academy of Sciences Beijing P. R. China; ^2^ College of Environment and Chemical Engineering Yanshan University Qinhuangdao P. R. China; ^3^ College of Chemical Engineering Beijing Institute of Petrochemical Technology Beijing P. R. China; ^4^ University of Chinese Academy of Sciences Beijing P. R. China

**Keywords:** agarose microspheres, agarose types, crosslinking degree, gel filtration chromatography, pore structure

## Abstract

Agarose microspheres with a controllable pore structure were manufactured by varying agarose types and crosslinking degrees. Various agarose could tailor the gel formation of microspheres matrix and thus affect the final pore structures. Small pores in microspheres could be fabricated by agarose with a higher molecular weight, which was demonstrated by the packed column with lower distribution coefficient (*K_av_*) values measured by gel filtration chromatography. Further, higher *K_av_* values also demonstrated that more and larger pores were formed with increasing the crosslinking degree of agarose microspheres. Either using agarose with a high molecular weight or increasing the crosslinking degree would finally lead to the enhancement of the flow rate during flow performance of packed column as necessary for improving separation efficiency. This provides a foundation for high‐resolution chromatography with a controllable separation range as beneficial for downstream process.

## 1 INTRODUCTION

**FIGURE 1:**
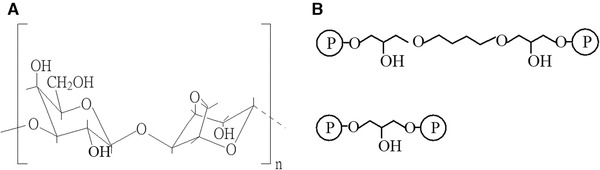
(A) Fundamental unit of agarose. (B) Agarose crosslinked with bis‐epoxides

The chromatographic separation of biomolecules has become typical steps in the biotechnology industry. Besides operation conditions of chromatography, chromatography media play a key role in the overall process performance. One of the most popular chromatography media is agarose‐based microspheres, due to its good properties as follows: (a) it is hydrophilic and insoluble; (b) it is chemically stable under the operation conditions; (c) it does not contain charged or ionogenic groups; (d) it has a good mechanical property; (e) it is easy to be functionalized; (f) it contains good pore structures for molecular sieving. Agarose is a kind of linear polysaccharide consisting of alternating residues of D‐galactose and 3, 6‐anhydro‐L‐galactose. The formulation of agarose into spherical microspheres, which by virtue of forming network bundles of polygalactan by hydrogen bonds, offers a high surface area beneficial for chromatography (Figure 1A). After being crosslinked and functionalized, agarose microspheres have achieved widely applications in chromatography, fixed bed, electrophoresis, biocatalyst, microbial growth and electrofocusing [1, 2, 3, 4].

Preparation of crosslinked agarose microspheres have been widely mentioned since the procedure was described by Hjertén [5]. The main methods include suspension gelation [5, 6] and spraying gelation [7, 8]. A new method called membrane emulsification technique was introduced to prepare agarose microspheres with narrow size distribution [9]. The basic procedure of these methods is to prepare the emulsion firstly by dispersing agarose solution into non‐polar organic solvent containing stabilizers under favorable conditions and then droplets are solidified by cooling the dispersion to the temperature below the gelling point of agarose, leading to the formation of agarose microspheres. To improve the chemical and physical properties of agarose microspheres, covalently crosslinking is carried out in an aqueous environment. Epichlorohydrin, bis‐epoxides and divinyl sulphone (DVS) are often used in the crosslinking procedure (Figure 1B) [10, 11, 12, 13, 14].

As one of the most important properties of chromatography media, the pore structure facilitates the transport of protein molecules through the gel network and has direct effects on the efficiency in chromatography. In gel filtration chromatography, pore structure of agarose microspheres has a close relation with its exclusion limit, and protein molecules having different sizes will pass the column at different times. Larger molecules cannot penetrate deeper into the pores of the microspheres and will leave the column first, and smaller molecules will leave the column later, and hence a separation of protein mixtures will be achieved. Also in adsorption chromatography, pore structure of agarose microspheres has great effects on its adsorption properties, for example, adsorption capacity, adsorption rate, etc. [15, 16, 17, 18, 19].The pores of agarose microspheres are formed from heterogeneous matrix region containing double helices arranged in bundles due to hydrogen bonds and both SEM analysis and chromatographic experiments are often performed to study the pore structure of agarose microspheres, the former gives a direct observation of the pore distribution and the latter is used to elucidate the permeation behavior of agarose microspheres [20, 21]. There are many factors affecting the pore structure of agarose microspheres, for example, properties of agarose, crosslinking agent, porogenic agent, etc [20, 22]. Firstly, the properties of agarose solution have a great influence on the formation of the pore structure and the pore size changes with the concentration of agarose solution [23]. Yet, the effects of agarose types, for example, the molecular structure, have not been reported. Secondly, crosslinking agent is significant to the pore structure; however, there are no reports of the effects of crosslinking degree as yet. Thirdly, the porogenic agent plays an important role in the pore structure and very large flow pores have been reported to be formed through a double emulsification procedure [24] or using granules of calcium carbonate as a porogen [25].

**PRACTICAL APPLICATION**

Agarose microspheres with a controllable pore structure were manufactured by varying agarose types and crosslinking degrees. Small pores in microspheres could be fabricated by agarose with a higher molecular weight, and more and larger pores were formed with increasing the crosslinking degree of agarose microspheres. Either using agarose with a high molecular weight or increasing the crosslinking degree would finally lead to the enhancement of the flow rate. Microspheres with controllable pore structures provide a foundation for wide applications in downstream process for downstream process.

Gel filtration chromatography experiments have been widely used for evaluation of the size exclusion effects of various solutes loaded to columns. Distribution coefficient (*K_av_*) is one of the most important parameters of chromatography media's and directly associated with both their pore structure and the solute's molecular size. Under certain conditions, the higher the *K_av_* value, the longer the distance passed for the solute, and also the greater the number of pores with same sizes to this solute distributed in chromatography media. Contrarily, the number is smaller. Also, *K_av_* values are closely related to separation ranges of gel filtration chromatography columns. If *K_av_* value is closer to 0, it represents that the solute can't enter into pores and reaches the maximum limit at this point. If *K_av_* value is closer to 1, it represents that smaller solutes can enter into pores and reached the minimum limit as peaks can't be well separated. Rich and well‐controlled pore structure is beneficial for broadening separation ranges and improving resolution, as very important for high‐resolution chromatography.

In the present work, agarose microspheres with different pore structures were manufactured and the effects of both agarose types and the crosslinking degree on the pore structure were discussed. SEM analysis, pressure‐flow rate experiments and chromatographic experiments were performed to study the properties of agarose microspheres and the results are useful for manufacturing agarose microspheres with a controllable pore structure for wide applications in downstream process. The effects on pore structure were considered during the preparation of high‐resolution chromatography microspheres and well‐controlled separation ranges were obtained by adjusting the crosslinking process.

## 2 MATERIALS AND METHODS

### 2.1 Materials

Agarose was purchased from Lonza (USA), Promega (USA) and Biowest (Spain), respectively. Blue Dextran (2000 kDa), thyroglobulin (660 kDa), BSA (66.12 kDa), RNA (13.7 kDa), and fluorescein isothiocyanate (FITC) were from Sigma (USA). rHBsAg were kindly provided by National Engineering Research Center for Biotechnology (China). QZT 4FF was kindly provided by Senhui Microspheres Tech (Suzhou) (Suzhou, Jiangsu, China). All other chemicals were of analytical grade and all solutions were made using Milli Q‐grade water (Millipore, USA).

### 2.2 Methods

#### 2.2.1 Viscosity of agarose solution

A total of 25 mL 6% w/v agarose solution was heated at 100°C under stirring and kept for 15 min. The viscosity of the solution was measured at different temperature in a range of 80°C to 55°C with a Brookfiled DV‐II+Pro viscometer (USA).

#### 2.2.2 Water absorbency of agarose

An amount of 1 g agarose was mixed with 20 mL water in a glass beaker overnight at 25°C and the mixture was then transferred to a glass filter‐funnel to filter the water. Water absorbency of agarose was calculated as follows:

$$\mathrm{Water}\;\mathrm{absorbency}\;(g/g)=\frac{20-m_{\mathrm{water}}-m_{0}}{m_{\mathrm{agarose}}}$$

where m_water_ was the amount of filtered water (g), m_0_ was the amount of water absorbed by the filter paper and represented as the blank value (g), m_agarose_ was the amount of agarose (g).

#### 2.2.3 Manufacture of agarose microspheres

Agarose microspheres were manufactured in two steps. Firstly, agarose microspheres were prepared according to the method of Hjertén [5]. A 6% w/v agarose solution was dissolved by boiling and used as water phase. Liquid paraffin containing Span 85 was heated to 60°C and used as oil phase. Then the water phase was added into the oil phase and the mixture was emulsified with stirring. After that, the emulsion was cooled down and agarose micropheres were washed thoroughly. Microspheres with a diameter range of 45–165 µm were obtained after sieving using various commercial supply types of agarose, that is, Lonza agarose, Promega agarose and Biowest agarose.

Secondly, agarose microspheres were crosslinked according to the method of Porath et al [11]. The whole crosslinking process was improved by using step by step technology as follows. 1st crosslinking: Agarose microspheres were mixed with acetone, epichlorohydrin and sodium hydroxide solution and stirred for 24 h at 40°C. After the reaction, microspheres were washed thoroughly. 2nd crosslinking: Agarose microspheres after 1st crosslinking were mixed with dimethyl sulfoxide and epichlorohydrin, sodium hydroxide solution and stirred for several hours at 40°C. After that cross‐linked microspheres were thoroughly washed. During 2nd crosslinking, a reaction time of 4 and 24 h was used, respectively. Microspheres with different crosslinking degrees were manufactured by adjusting the crosslinking procedure mentioned above.

The morphology of wet agarose microspheres was recorded by an optical microscope (EVOS XL, Advanced Microscopy Group, USA). The size of agarose microspheres was measured by laser diffraction using Mastersizer 2000E (Malvern Instruments, UK). The surface morphology of agarose microspheres after being dried was observed by scanning electron microscope (SEM, JEM‐6700F, JEOL, Japan).

#### 2.2.4 Pressure‐flow rate test

For testing the rigidity of microspheres, pressure‐flow rate test was performed. A column (10 cm × 1.0 cm id) was packed with agarose microspheres and collected to Protein Purification System (Senhui Microspheres Tech (Suzhou)) (Suzhou, Jiangsu, China). Firstly, the microspheres were settled by gravity until 1 cm of particle‐free fluid space was formed. A low pressure was then applied to start until the bed height was stabilized. To obtain a pressure‐flow rate curve, various flow rates were set and the bed height was stabilized after a few minutes, then the pressure drop was recorded by a pressure gauge. The linear flow rate was calculated as follows:

$$\mathrm{Flow}\;\mathrm{rate}\left(\mathrm{cm}/h\right)=\frac{V_{x}}{0.785\times t}$$

where *V_x_* was the volume of the water flowed out of the column (mL), *t* was the period of time (h), 0.785 was the coefficient representing the cross‐sectional area of the column (cm^2^). The pressure‐flow rate data of the empty column was determined and set as the blank value.

#### 2.2.5 Chromatographic performance

A column (30 cm × 1.0 cm id) was packed with agarose microspheres and was equilibrated with the running buffer (50 mM phosphate, 0.15 M NaCl, pH 7.0) until a stable baseline was achieved. 100 µL of the standard protein (2 mg/mL) dissolved in the running buffer was introduced into the column and the same buffer was used for elution. Thyroglobulin, BSA and RNA were used as standard proteins, respectively. The detection wavelength was set at 280 nm and fractions corresponding to each elution peak were pooled. All of the chromatographic experiments were performed on Protein Purification System at room temperature and a flow rate of 0.5 mL/min was maintained throughout. Each chromatographic run was performed in duplicate. *K_av_* values were calculated as follows:

$$K_{\mathrm{av}}=\frac{V_{r}-V_{0}}{V_{t}-V_{0}}$$

where *V_r_* was the peak retention volume (mL), *V_0_* was the extraparticle volume (mL) determined by Blue Dextran 2000 and *V_t_* was the included or total mobile phase volume (mL) determined by acetone.

rHBsAg was loaded into a column (30 cm × 1.0 cm id) packed with QZT 4FF equilibrated with the running buffer (20 mM phosphate, 0.15 M NaCl, pH 9.6). 500 µL of rHBsAg (2 mg/mL) dissolved in the running buffer was used and the chromatographic procedure was the same as above. The detection wavelength was set at 280 nm and fractions corresponding to each elution peak were pooled. Each chromatographic run was performed in duplicate and the chromatogram was recorded.

## 3 RESULTS AND DISCUSSION

**FIGURE 2:**
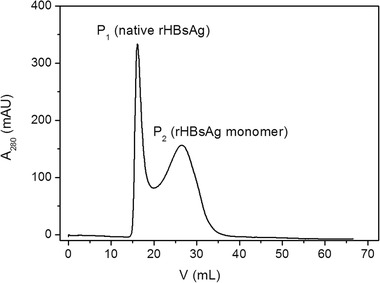
Gel filtration chromatogram of Hans‐HBsAg on QZT 4FF column (30 cm × 1.0 cm id)

In chromatography, protein components are separated by their column retention properties and the pore structure has a great impact on protein separation. Proteins with proper molecular weights can diffuse into the inner part of chromatography media and are evenly distributed in the microspheres. It's difficult for large molecules to diffuse into the pores and too large molecules can't completely enter, so they flow out the column early. On the contrary, small molecules can enter into the inner pores and flow out the column later. Here, gel filtration chromatography experiments were performed and rHBsAg derived from *Hansenula polymorpha* yeast cell (Hans‐HBsAg) was loaded into the column packed with QZT 4FF (commercial agarose microspheres used for gel filtration chromatography). rHBsAg used here was composed of two parts, one was native rHBsAg and the other was its monomer, with an average molecular weight of about 3000 kDa and 24 kDa, respectively. In Figure 2, there were two peaks as P_1_ represented native rHBsAg and P_2_ represented rHBsAg monomer, respectively. According to the separation ranges of QZT 4FF, that is, 60–20000 kDa, the molecular size of rHBsAg was close to the maximum exclusion limit of agarose‐based microspheres and it can hardly enter into their inner pores, whereas its monomer can. This paper focused on the effects of both the types of agarose and crosslinking degrees on pore structure of chromatography media and their separation ranges.

### 3.1 Characterization of agarose

**FIGURE 3:**
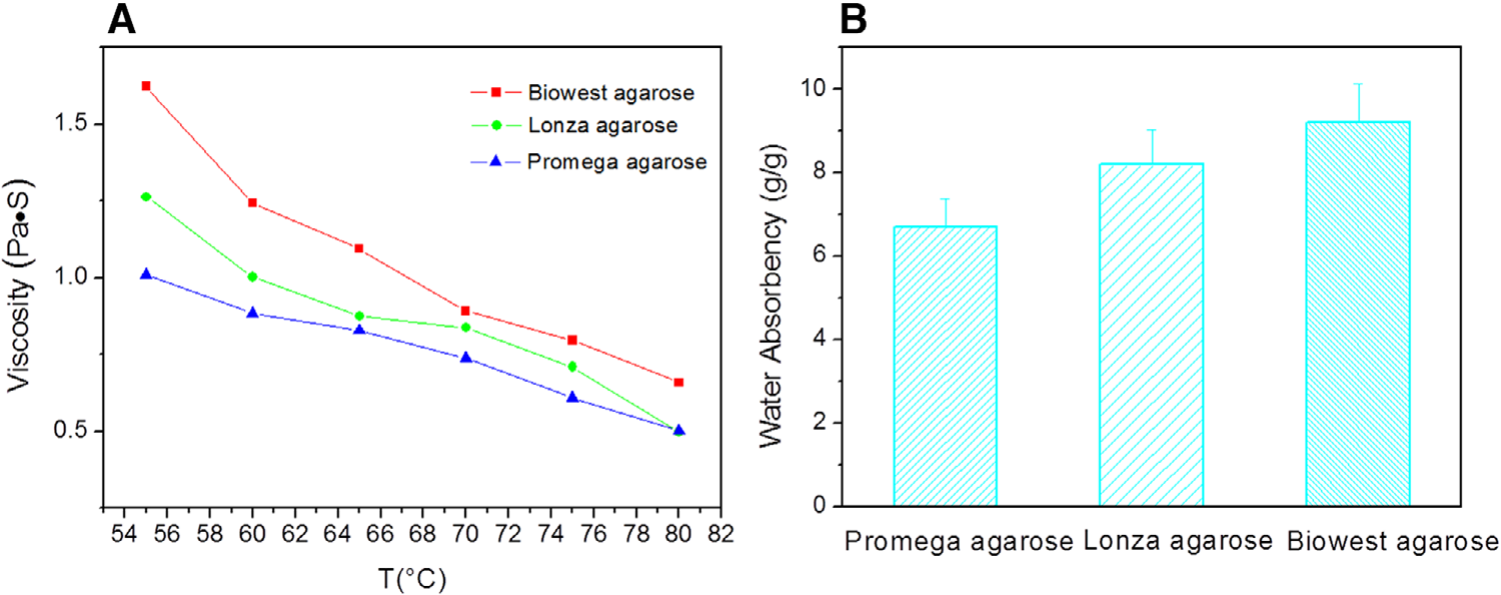
(A) The viscosity of agarose solution as a function of temperature. (B) Water absorbency of various types of agarose

The effects of temperature on the viscosity of agarose solution were shown in Figure 3A. The viscosity decreased with the temperature, due to that the molecular chains became more flexible when heated. The viscosity of Biowest agarose was the highest, followed by Lonza agarose, Promega agarose was the lowest. The viscosity of agarose solution had a great relation with its molecular structure and a Mark‐Houwink equation for agarose was used to determinate the correlation between the intrinsic viscosity and the weight‐average molecular weight:

$$\left[\eta \right]=0.07\;M^{0.72}$$

where [*η*] was mL/g and M was the weight‐average molecular weight. For agarose solution with a higher viscosity, a higher molecular weight was possessed [26]. Therefore, Biowest agarose was supposed to have the longest molecular chain, then went for Lonza agarose and Promega agarose had the shortest one.

Water molecules participated in hydrogen bonds to form stable double helix in agarose gel and water absorbency of agarose had a close relation with its molecular structure due to that more water were needed to form stable helix for agarose molecules with longer chains [27]. In Figure 3B, water absorbency of Biowest agarose was the highest, followed by Lonza agarose, Promega agarose was the lowest, a same order with the viscosity of agarose solution, also indicating that the molecular chains of Biowest agarose were the longest and those of Promega agarose were the shortest.

### 3.2 Characterization of agarose microspheres

**FIGURE 4:**
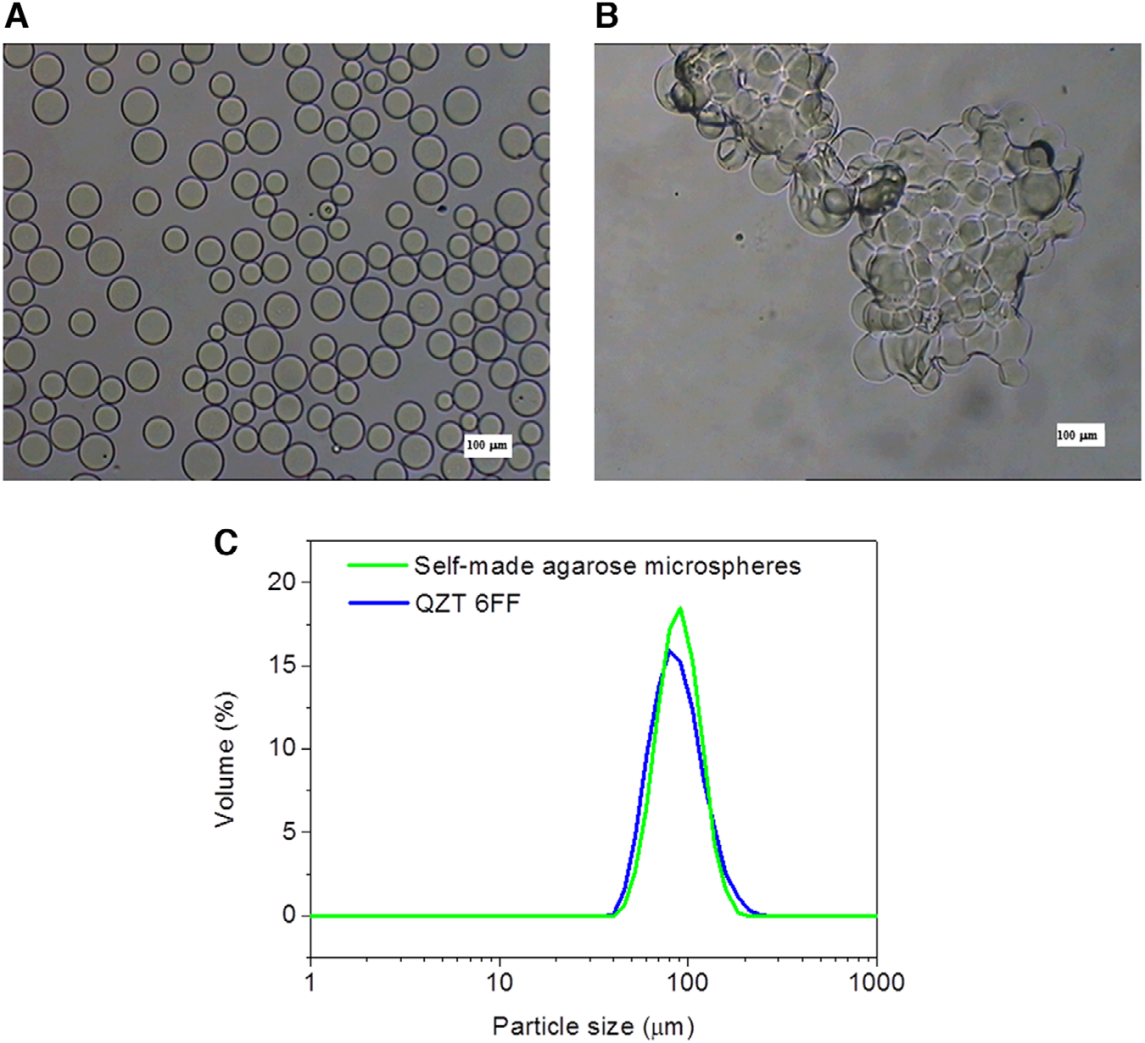
Optical microscopic photographs of (A) agarose microspheres prepared using Lonza agarose, (B) non‐crosslinked, (C) the particle size distribution of self‐made agarose microspheres compared with commercial microspheres

**FIGURE 5:**
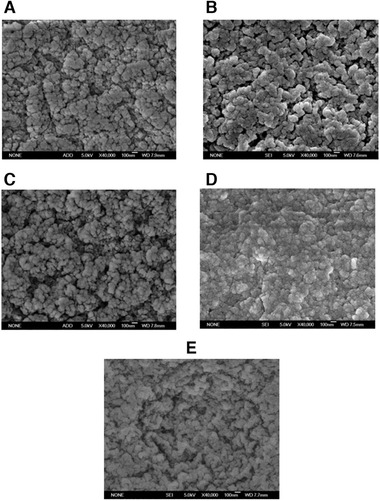
SEM photographs of agarose microspheres manufactured by 2nd crosslinking with a reaction time of 4 h using (A) Biowest agarose, (B) Lonza agarose, (C) Promega agarose and by (D) 1st crosslinking with a reaction time of 24 h and (E) 2nd crosslinking with a reaction time of 24 h using Lonza agarose (40 000 ×)

During the manufacture of agarose microspheres, processing conditions were optimized including the property of oil phase, the stirring speed, the type and the amount of the stabilizer, the ratio of the amounts of the water and oil phases, and the microspheres with a diameter range from 45 to 165 µm were collected. In Figure 4, the morphology and particle size of agarose microspheres prepared using Lonza agarose were recorded, respectively. Agarose microspheres had a perfectly spherical shape and the average diameter was about 90 µm, as comparable to that of commercial beads size distribution (Figure 4A and C). Also, non‐crosslinked agarose microspheres after freeze‐drying cannot reconvert after incubation in water, probably because that its pore structure was destroyed after freeze‐drying, while crosslinked agarose microspheres reconverted well, because that the rigidity of the microspheres was high enough to protect the pore structure (Figure 4B and A). In Figure 5, microspheres manufactured using various types of agarose and crosslinking degrees were observed by SEM, respectively, to study the effects of agarose types and the crosslinking degree on the pore structure and the results were as follows.

#### 3.2.1 Agarose types

Agarose microspheres from various types of agarose were obtained after 2nd crosslinking with a reaction time of 4 h and the integral morphology of the microspheres was well kept after freeze‐drying except for a little shrinkage (Figure 5A–C representing Biowest agarose, Lonza agarose and Promega agarose, respectively). Evidently, there were a lot of pores distributed homogeneously on the surface of the microspheres. Although the porosity and its distribution were almost the same generally, more pores were distributed on the surface since the volume was larger at the surface than that in the center of the microspheres [17]. The pore morphology of the microspheres from various types of agarose was very different, and the pore size of Biowest agarose microspheres was the smallest, while that of Promega agarose microspheres was the largest. Agarose with a higher molecular weight tended to form microspheres with smaller pores, while that with a looser one tended to form microspheres with larger pores.

#### 3.2.2 Crosslinking degree

Agarose microspheres were manufactured with different crosslinking degrees and their pore structure was studied. Crosslinking played an important role in the pore structure of agarose microspheres. Figure 5D, B and E represented agarose microspheres were manufactured by 1st crosslinking with a reaction time of 24 h, 2nd crosslinking with a reaction time of 4 h and 24 h, respectively. There were not obvious pores on the surface of the microspheres by 1st crosslinking with a reaction time of 24 h, probably due to that the rigidity of these microspheres was not high enough to keep their morphology and the pores distorted after freeze drying (Figure 5D). More pores appeared on the surface by 2nd crosslinking with a reaction time of 4 h and 24 h (Figure 5B and E), respectively, and one of the most important reasons was that the mechanical strength of the gel structure was high enough after crosslinking to endure the deformation caused by freeze drying.

### 3.3 Rigidity of agarose microspheres

The effects of agarose types and the crosslinking degree on the rigidity of agarose microspheres were studied by pressure‐flow rate analysis (which is a measure of the rigidity of the microspheres [13]).

#### 3.3.1 Agarose types

**FIGURE 6:**
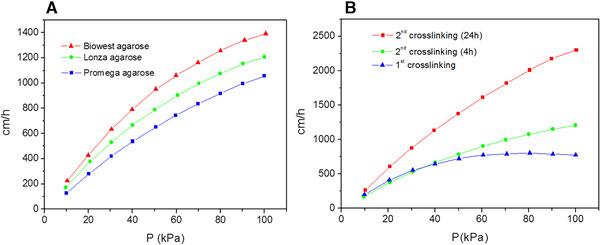
The pressure‐flow rate curves of agarose microspheres with (A) various types of agarose and (B) different crosslinking degrees

The pressure‐flow rate curves of the microspheres from various types of agarose after 2nd crosslinking with a reaction time of 4 h were shown in Figure 6A. All of these three types of agarose microspheres displayed a linear pressure‐flow rate behavior with a linear flow rate up to 1000 cm/h corresponding to a pressure drop of 100 kPa, and the flow rate increased with the pressure drop. Under the same pressure drop, the flow rate of Biowest agarose was highest, followed by Lonza agarose, Promega agarose was the lowest. So why the microspheres from agarose with a lower molecular weight, for example, Promega agarose, had larger pores as shown by SEM and yet had a lower flow rate? A reasonable explanation was that a reversible deformation occurred with Promega agarose microspheres during the pressure‐flow rate test and hence the pores became smaller, demonstrated by the fact that there was an obvious compression in the column during the test as was recovered finally. Accordingly, its permeability was worse and a lower flow rate was got under the same pressure drop. The rigidity of the microspheres has a great influence on the degree of microspheres deformation, affected by agarose types and a highly aggregated gel network was achieved from agarose with a higher molecular weight, leading to a higher rigidity and less deformation. On the contrary, the microspheres with a lower rigidity were formed from agarose with a lower molecular weight and hence more reversible deformation occurred under the same pressure [27].

#### 3.3.2 Crosslinking degree

The pressure‐flow rate curves of agarose microspheres with different crosslinking degrees were shown in Figure 6B. The flow rate of the microspheres was gradually improved with the crosslinking degree, indicating that its rigidity was greatly improved by crosslinking. Non‐crosslinked agarose microspheres had a very weaker rigidity and cannot endure a higher pressure than 30 kPa. After 1^st^ crosslinking, the maximum pressure that the microspheres could endure was up to 60 kPa within a linear flow rate up to 750 cm/h, and after that the flow rate didn't increased any more. After 2nd crosslinking, the rigidity of agarose microspheres was further improved with a linear flow rate up to 1250 cm/h and 2250 cm/h with a crosslinking time of 4 h and 24 h, respectively. Crosslinking is capable of locking or tying the chains together and thereby increasing the rigidity of the microspheres. The great increase in the rigidity of agarose microspheres made it possible to apply much higher pressure to the columns and hence the flow‐rate increased accordingly, extremely meaningful in chromatography process.

### 3.4 Chromatographic property

In gel filtration chromatography, the permeation behavior of agarose microspheres packed into a column has a close relation with its pore structure and can be studied through *K_av_* values. Here, agarose microspheres were packed into a column for gel filtration chromatography and protein samples with a range of molecular weights were injected and eluted, and *K_av_* value of each sample was calculated accordingly. The effects of agarose types and the crosslinking degree on *K_av_* values were investigated.

#### 3.4.1 Agarose types

**FIGURE 7:**
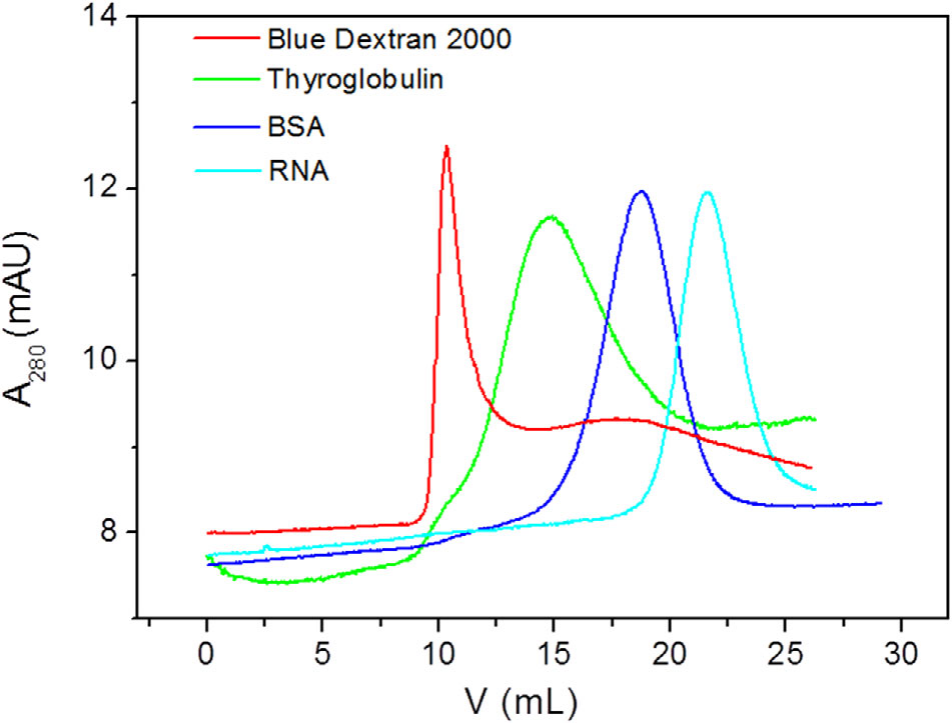
Chromatogram obtained with standard proteins after passage through a column packed with Biowest agarose microspheres manufactured by 2nd crosslinking with a reaction time of 4 h

Protein samples were loaded on the column packed with agarose microspheres and eluted in the order for molecular sieving as shown in Figure 7. All of the microspheres were manufactured after 2nd crosslinking with a reaction time of 4 h. From Table 1, *K_av_* values of samples from columns packed with microspheres from various types of agarose were different except for blue dextran 2000 and acetone, indicating that the pore structure of these microspheres was different. *K_av_* values from Biowest agarose microspheres were lowest except for RNA, followed by Lonza agarose microspheres, and the values from Promega agarose microspheres were highest. For a certain sample, a lower *K_av_* value was due to that the sample solute was hindered by smaller pores and hence flew through the column more quickly. The results confirmed that the pores formed in Biowest agarose microspheres were smallest, followed by Lonza agarose microspheres and Promega agarose microspheres, respectively, and also proved by SEM analysis. The exception that *K_av_* of RNA from Biowest agarose microspheres was higher than that from Lonza agarose microspheres was probably because there were more small pores distributed in Biowest agarose microspheres just for RNA penetration. The fact that smaller pores are formed using agarose with a high molecular weight has also been reported for carrageenan, probably because longer molecular chains are easily to be twisted during the gelation process leading to formation of smaller pores [28, 29].

**TABLE 1:** K_av_ values of standard proteins loaded on the column packed with agarose microspheres manufactured using various types of agarose

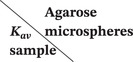	Biowest agarose microspheres	Lonza agarose microspheres	Promega agarose microspheres
Blue Dextran 2000	0	0	0
Thyroglobulin	0.339	0.371	0.485
BSA	0.630	0.653	0.722
RNA	0.845	0.839	0.882
Acetone	1	1	1

#### 3.4.2 Crosslinking degree

To study the effects of the crosslinking degree on chromatographic property, agarose microspheres were crosslinked with different procedures using Lonza agarose. Table 2 showed that *K_av_* values from columns increased with the crosslinking degree of the microspheres, due to that more and larger pores were formed with the increase of the crosslinking degree and hence the retention volumes of samples increased. The crosslinking bonds may be formed in intra‐ or inter‐helical regions and the porosity of the microspheres had a strong dependence on the length of the crosslinking agent and also on the crosslinking degree. Proteins were more retarded on the column packed with crosslinked microspheres than that with non‐crosslinked microspheres, indicating that agarose microspheres became more porous after crosslinking, also mentioned by Porath et al [14] finding that agarose microspheres crosslinked with divinyl sulphone (DVS) contained considerably increased porosity, probably because crosslinking promoted new formation of the entanglement of molecular chains leading to more porous gel structure.

**TABLE 2:** K_av_ values of standard proteins loaded on the column packed with agarose microspheres with different crosslinking degrees using Lonza agarose

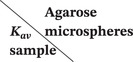	Agarose microspheres after 1st crosslinking	Agarose microspheres after 2nd crosslinking (4 h)	Agarose microspheres after 2nd crosslinking (24 h)
Blue Dextran 2000	0	0	0
Thyroglobulin	0.324	0.371	0.410
BSA	0.523	0.653	0.670
RNA	0.718	0.839	0.851
Acetone	1	1	1

The estimation of the pore size of the gel materials based on processing parameters is of great importance for manufacture of adsorbent particles. The pore size of dextran microspheres can be well explained using the following model [30]:

$$\xi =0.071\cdot {\left(\nu_{2,s}\right)}^{-1/3}\cdot {M_{c}}^{1/2}$$

where ξ is the mesh size in nanometers representing the pore size of dextran microspheres, ν_2,s_ is the volume fraction of dextran and M_c_ is the average molecular weight between the crosslinks in kg/mol. At a fixed ν_2,s_ (i.e., dextran volume fraction is constant) for microspheres with same dextran content, the pore size increases with M_c_. In our results, the effects of processing parameters on the pore size of agarose microspheres were partially consistent with this model. On one hand, a higher M_c_ was got by the crosslinking reaction, and larger pores are formed as shown by the experimental results as consistent with the above model. On the other hand, a higher M_c_ was also achieved with agarose with a high molecular weight, and yet smaller pores are formed as contrary to the model. The reason for this deviation was probably due to the difference of physical properties between agarose and dextran needing to be studied further.

## 4 CONCLUDING REMARKS

The effects of processing parameters on the pore structure of agarose microspheres are highly meaningful for manufacture of microspheres with a controllable pore structure. Both agarose types and the crosslinking degree had great effects on the manipulation of the pore structure. Smaller pores were formed in the microspheres from agarose with a higher molecular weight while more and larger pores were formed with increasing the crosslinking degree within a certain range. The microspheres with a good rigidity were manufactured using agarose either with a higher molecular weight or by increasing the crosslinking degree. The relation between these two processing parameters and the pore size of agarose microspheres was partially consistent with a pore size model of dextran microspheres. During the preparation of high‐resolution chromatography microspheres, the crosslinking procedure was adjusted and there were significant changes in separation ranges of columns as microspheres with high crosslinking degrees are more suitable for separation of solutes with low molecular weights. This lays a good foundation for development of high‐resolution chromatography with well‐controlled separation ranges.

## CONFLICT OF INTEREST

The authors have declared no conflict of interest.
